# Fecal *Bifidobacterium* Serves as a Predictor of Postoperative Recurrence After Neoadjuvant Chemotherapy in Pancreatic Cancer

**DOI:** 10.1016/j.gastha.2025.100779

**Published:** 2025-08-28

**Authors:** Ayaka Takaori, Tsukasa Ikeura, Daisuke Hashimoto, Motonobu Maruo, Masatoshi Ikeda, Takashi Ito, Koh Nakamaru, Masataka Masuda, Shinji Nakayama, Hidetaka Miyazaki, Kazuki Matsumura, So Yamaki, Tomoyo Yasuda, Masashi Kanai, Shohei Akagawa, Shoji Tsuji, Koichiro Higasa, Sohei Satoi, Makoto Naganuma

**Affiliations:** 1 Third Department of Internal Medicine, Kansai Medical University, Osaka, Japan; 2 Department of Pancreatobiliary Surgery, Kansai Medical University, Osaka, Japan; 3 Department of Clinical Oncology, Kansai Medical University, Osaka, Japan; 4 Department of Pediatrics, Kansai Medical University, Osaka, Japan; 5 Department of Genome Analysis, Institute of Biomedical Science, Kansai Medical University, Osaka, Japan; 6 Division of Surgical Oncology, University of Colorado Anschutz Medical Campus, Aurora, Colorado

**Keywords:** Gut Microbiota, Neoadjuvant Therapy, Pancreatic Ductal Adenocarcinoma, *Bifidobacterium*

## Abstract

**Background and Aims:**

To date, neoadjuvant chemotherapy (NAC) is a well-accepted therapeutic strategy for patients with resectable (R) and borderline resectable (BR) pancreatic ductal adenocarcinoma (PDAC). We previously reported that the relative abundance of *Bifidobacterium* in the gut microbiota is associated with a more favorable pathological response to NAC in R/BR-PDAC. In the current study, we evaluated the association between pretreatment gut microbiota and postoperative prognosis in patients with R/BR-PDAC who underwent pancreatectomy after NAC.

**Methods:**

In this prospective observational study, we analyzed 42 patients with R/BR-PDAC who underwent pancreatic resection following NAC with gemcitabine plus S-1 between 2020 and 2022 at Kansai Medical University Hospital. Stool samples were collected at PDAC diagnosis for microbiota analysis using 16S RNA gene sequences. In 19 genera accounting for ≥ 1% in relative abundance, the relationship between the microbiota profile and recurrence-free survival (RFS) and overall survival was examined using the Kaplan–Meier method and the Cox proportional hazards model.

**Results:**

The median postoperative observation period was 24 months. During the observation period, 23 patients (55%) experienced recurrence, and 10 patients (24%) died of PDAC. RFS was significantly favorable in patients in the high-*Bifidobacterium* group (relative abundance ≥ 4%) compared to patients in the low-*Bifidobacterium* group (<4%). Furthermore, multivariable analysis revealed high-*Bifidobacterium* and pathological metastasis of lymph node were significantly associated with RFS (hazard ratio 0.37; 95% confidence interval 0.14–0.97, *P* = .042). Regarding overall survival, there was no significant association with gut microbiota, including *Bifidobacterium* genus (hazard ratio 0.23; 95% confidence interval 0.03–1.50), in the multivariable analysis.

**Conclusion:**

The relative abundance of *Bifidobacterium* in the gut microbiota at diagnosis may be a predictor of postoperative recurrence in patients with R/BR-PDAC treated with NAC.

## Introduction

Pancreatic ductal adenocarcinoma (PDAC) has a poor prognosis, with a 5-year survival rate of 12%.^1^ Treatment modalities for PDAC have been modified to optimize patient outcomes. Currently, in resectable (R) and borderline resectable (BR) PDAC, several studies have reported that the overall survival (OS) of patients who receive neoadjuvant chemotherapy (NAC) before curative resection is more favorable than that of patients undergoing upfront surgery,^2^, ^3^, ^4^ leading to the widespread use of NAC in patients with R/BR-PDAC, according to guidelines.^5^ However, no biomarkers are available to predict the therapeutic effects of NAC before therapy.

The association between the gut microbiota and pathological conditions of various diseases is widely acknowledged. Accumulating evidence suggests that gut bacteria could influence the response to treatment and prognosis in PDAC. Mitsuhashi et al. observed that the presence of *Fusobacterium* in human pancreatic cancer specimens was correlated with a significant increase in cancer-specific mortality^6^ In addition, studies have recently revealed a relationship between intratumoral microbiota and clinical outcome.^7^, ^8^, ^9^ Riquelme et al. reported that patients with long-term survival exhibited higher alpha diversity in the tumor microbiome than those with short-term survival.^8^ In this study, a unique intratumoral bacterial composition characterized by *Pseudoxanthomonas, Streptomyces*, *Saccharopolyspora*, and *Bacillus clausii* was identified as a highly predictive marker of long-term survival; these specific genera in the tumor were correlated with CD8 positive (CD8^+^) T cell tissue densities, suggesting that bacteria might contribute to the antitumor immune response by enhancing the requirement and activation of CD8^+^ T cells. Meanwhile, in chemotherapy for PDAC with metastasis, administering antibiotics before or after beginning first-line chemotherapy is associated with improved survival, indicating that the microbiota might modulate therapeutic efficacy in patients with PDAC^10^ Based on these results, microbiome research may lead to the development of novel biomarkers and therapeutic approaches to improve the prognosis of patients with PDAC.

Recently, we evaluated the impact of NAC for R/BR-PDAC on the gut microbiota and revealed that the diversity of the gut microbiota was not decreased by NAC.^11^ Further, we reported that a higher relative abundance of *Bifidobacterium* in the stool at the time of diagnosis showed a more favorable pathological response to NAC.^11^ However, whether pretreatment gut microbiota is associated with postoperative outcomes, which are the most critical factors in treating cancer, in patients with R/BR-PDAC receiving resection following NAC remains unclear. Thus, this study aimed to reveal the microbial signatures associated with the outcomes of R/BR-PDAC resection after NAC. The results of our study could contribute to the identification of predictive microbiota-based biomarkers for prognosis and the development of novel therapeutic options to improve PDAC prognosis.

## Materials and Methods

### Patients

In this prospective observational study, we analyzed 42 patients with R/BR-PDAC who underwent pancreatic resection after NAC with gemcitabine plus S-1 (GS) between September 2020 and December 2022 at Kansai Medical University Hospital. Eighteen of the 42 patients were included in a previous study.^11^

PDAC was diagnosed based on the pathological findings using either endoscopic retrograde cholangiopancreatography or endoscopic ultrasound-guided tissue acquisition.

The resectability of PDAC was radiologically determined as R or BR, according to the National Comprehensive Cancer Network guidelines.^12^ Radiological examination was performed using cine-imaging multidetector computed tomography with an Aquilion Computed Tomography system (Toshiba Medical Systems, Tochigi, Japan) during the patient’s initial visit to the hospital.^4^

This prospective study was approved by the Ethics Committee of Kansai Medical University (No. 2020208). Written informed consent was obtained from all patients before enrollment in the study.

### Collection of Clinical Data

We collected the clinical data of the 42 patients, including their demographic profile, medical history, family history, alcohol and smoking habits, laboratory data, radiological findings, chemotherapy (NAC and adjuvant therapy), adverse events, histopathological findings of the resected specimens, and outcomes. TNM staging was based on the 8th edition of the Union for International Cancer Control stage classification.^13^ Diabetes mellitus was defined as an HbA1c of 6.5% or the use of glucose-lowering medications at the time of registration.^14^

Nutritional status was assessed using the body mass index, serum albumin level, prognostic nutrition index (PNI), and neutrophil-to-lymphocyte ratio (NLR). PNI was calculated as follows: PNI score = 10 × serum albumin value (g/dL) + 0.005 × total lymphocyte count (per mm^3^).^15^ NLR was calculated as the ratio of the derived neutrophil and lymphocyte counts (per mm^3^).^16^

### DNA Extraction From Stool Samples and 16S Ribosomal RNA (rRNA) Gene Sequencing for Taxonomic Annotation

Because medications such as chemotherapy can alter the gut microbiota,^17^ stool samples were collected at the time of diagnosis of R/BR-PDAC. The samples were immediately transferred to −80 °C and stored until further processing.

To identify the bacterial composition of the samples, 16S rRNA amplicon sequencing was performed. Sequencing of the 16S rRNA gene was performed by Macrogen Japan, Inc (Tokyo, Japan). Sequencing libraries were prepared according to the Illumina 16S metagenomic sequencing library protocol to amplify the V3 and V4 regions.

Initially, DNA was extracted from stool samples using a NucleoSpin Microbial DNA Kit (Macherey-Nagel, Düren, Germany). DNA was isolated from stored stool samples using a Feces Collection Kit (Techno Suruga Lab, Shizuoka, Japan) via mechanical disruption using a bead beat and a silica membrane spin column. The extracted DNA was purified using an Agencourt AMPure XP (Beckman Coulter, Brea, CA). DNA was amplified using PCR. Sequence reads were determined using an Illumina MiSeq System (Illumina, San Diego, CA) and were imported into Quantitative Insights Into Microbial Ecology version 2 pipeline (version 2021.12) for analysis of bacterial identification.^18^ Chimeric and low-quality reads were filtered out during denoising process. High-quality reads with quality scores greater than 20 were selected and clustered into amplicon sequence variants using Divisive Amplicon Denoising Algorithm 2. The identified amplicon sequence variants were aligned to the SILVA reference database for the taxonomic classification of the bacterial communities. Features containing host DNA, such as mitochondria and chloroplasts, were excluded.

### Analysis of the Association Between Microbiome and Prognosis

From relative abundance data obtained with 16S rRNA sequencing, we calculated the mean relative abundance of each genus in all the patients included in the current study and identified bacterial genera with relative abundance ≥ 1% of all taxa that were most likely to be involved with the prognosis of resected R/BR PDAC, as reported previously.^11^^,^^19^ Next, we divided the patients into 2 groups (high and low relative abundance in gut microbiota) based on the median value of the relative abundance in each of the 19 genera and generated Kaplan–Meier curves of the 2 groups to compare recurrence-free survival (RFS) and OS. Subsequently, we evaluated the differences between the high and low relative abundances of each genus using the log-rank test to identify candidate microbial genera for predicting postoperative RFS and OS.

We conducted linear discriminant analysis effect size (LEfSe) using data of all bacterial genera identified with 16s rRNA sequencing to identify microbial genera related with recurrence within 12 months after resection.

### NAC and Adjuvant Chemotherapy

All the patients underwent NAC with GS therapy. Gemcitabine was administered at 1000 mg/m^2^ on days 1 and 8, and S-1 was administered orally at a dose appropriate for body surface area (BSA) 2 times daily on days 1-14 of a 21-day cycle (BSA < 1.25 m^2^, 40 mg; BSA 1.25–1.5 m^2^, 50 mg; BSA > 1.50 m^2^, 60 mg). All patients underwent surgical resection of PDAC within 8 weeks after NAC. As adjuvant chemotherapy, all patients underwent S-1 therapy for 6 months. The relative dose intensity (RDI) was calculated according to previous studies.^20^^,^^21^ The average dose of Gemcitabine received per week by the patient was divided by the expected dosage specified in the standard regimen.

Adverse events during NAC and adjuvant chemotherapy were evaluated by scoring their severity based on the Common Terminology Criteria for Adverse Events (version 4.0).

### Statistical Analysis

Continuous variables are expressed as medians (interquartile ranges). Continuous variables and proportions were compared using the Wilcoxon rank-sum and chi-square tests, respectively. RFS and OS were estimated using the Kaplan–Meier method. RFS and OS were defined as the time intervals from PDAC resection to the date of disease recurrence and from the start of PDAC treatment to the date of death or last follow-up, respectively. The comparison of RFS and OS in the Kaplan–Meier curves was performed using a log-rank test without false discovery rate correction. Univariable and multivariable analyses were performed using Cox proportional hazards model, a time-to-event analysis method, to identify the predictive factors for prognosis after resection. Multivariable analysis was carried out using age, gender, and parameters with *P* < .1 from univariable analysis. As no patients died without recurrence in the cohort, a competing risk model was not needed to analyze these data. Statistical significance was set at *P* < .05. Statistical analyses were performed using JMP Pro software (version 17.2.0; SAS Institute, Inc, Cary, NC).

## Results

### Patients’ Characteristics

The clinical characteristics of the patients are summarized in Table 1. Fifteen (36%) of the 42 patients were male, and the median age was 72.5 years. PDAC tumors were located in the pancreatic head in 31 patients (74%) and the pancreatic body/tail in 11 patients (26%). Regarding the resectability classification, 37 patients (88%) had R-PDAC, and 5 (12%) had BR-PDAC.

**Table 1:** Details of 42 Patients at Diagnosis of Pancreatic Ductal Adenocarcinoma

	N = 42
Male, n (%)	15 (36)
Age, median (IQR), y	72.5 (66.8-77)
Body mass index, median (IQR)	21.9 (20–24.4)
Albumin, median (IQR), g/dL	4.2 (4–4.5)
Prognostic nutritional index, median (IQR)	50.0 (45.4–53.3)
Neutrophil-to-lymphocyte ratio, median (IQR)	0.39 (0.31–0.51)
CA19-9, median (IQR), U/mL	40.8 (4-2920)
Current smoker, n (%)	14 (33)
Diabetes mellitus, n (%)	16 (38)
Family history of pancreatic cancer, n (%)	8 (19)
Oral medication	
Proton pump inhibitor, n (%)	19 (45)
Probiotics, n (%)	3 (7)
Antibiotics, n (%)	0 (0)
Tumor location	
Head, n (%)	31 (74)
Body/Tail, n (%)	11 (26)
Resectability classification	
Resectable PDAC, n (%)	37 (88)
Borderline resectable PDAC, n (%)	5 (12)

IQR, interquartile range; PDAC, pancreatic ductal adenocarcinoma.

### Treatment of PDAC and Prognosis

The details of the treatment of pancreatic cancer in the patients enrolled in this study are shown in Table 2. All patients received GS as NAC. The median RDI in the NAC group was 72.9%. Regarding adverse events by NAC (Common Terminology Criteria for Adverse Event Grade ≥ 3), no patients suffered from severe adverse events induced by gastrointestinal toxicities of NAC, such as diarrhea, nausea, and vomiting. However, 20 patients (48%) experienced neutropenia, which was resolved conservatively. The pathological stage of the resected PDAC was Stage IA in 8 patients (19%), Stage IB in 13 (31%), and IIB in 21 (50%). Regarding residual tumor status, R0 resection was performed in all patients. After pancreatectomy, adjuvant therapy with S-1 was administered to all patients for 6 months. The median follow-up period after the surgical resection was 24 months. After pancreatectomy, 23 patients (55%) experienced recurrence, and 10 patients (24%) died of the primary disease due to cancer recurrence.

**Table 2:** Summary of Treatment and Prognosis

	N = 42
Regimens of NAC	
Gemcitabine plus S-1, n (%)	42 (100)
Relative dose intensity of NAC, median (IQR), %	72.9 (56.6–85.7)
Adverse effect by NAC (CTCAE Grade ≥3)	
Neutropenia, n (%)	20 (48)
Diarrhea, n (%)	0 (0)
Stomatitis, n (%)	0 (0)
Pancreatic resection	
Pancreaticoduodenectomy, n (%)	31 (74)
Distal pancreatectomy, n (%)	11 (26)
Reduction rate of CA19-9 level before and after NAC	
≥50%, n (%)	13 (31)
<50%, n (%)	29 (69)
Pathological stage	
IA, n (%)	8 (19)
IB, n (%)	13 (31)
IIB, n (%)	21 (50)
Residual tumor status	
R0, n (%)	42 (100)
Postoperative complication	
Postoperative pancreatic fistula, n (%)	5 (12)
Delayed gastric emptying, n (%)	1 (2)
Post pancreatectomy infections, n (%)	2 (5)
Postoperative cholestasis, n (%)	3 (7)
Post pancreatectomy hemorrhage, n (%)	3 (7)
Adjuvant therapy	
S-1, n (%)	42 (100)
Median follow-up period after resection, median (IQR), mo	24 (18-31)
Postoperative recurrence, n (%)	23 (55)
Mortality, n (%)	10 (24)

CTCAE, Common Terminology Criteria for Adverse Events; IQR, interquartile range.

### Gut Microbiota in Patients Included in the Current Study

**Figure 1:**
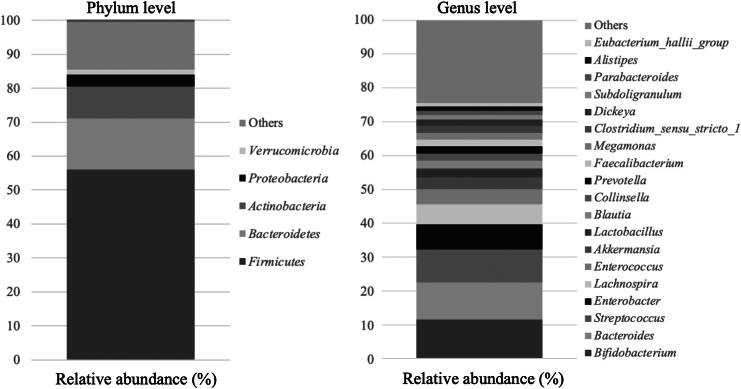
Microbiota composition in 42 patients Stacked bar plots show the mean relative abundances.

The distribution of the gut microbiota at the phylum and genus levels in 42 patients is shown in Figure 1. At the phylum level, Firmicutes were the most enriched in the cohort, which was consistent with the previous study.^10^ Among 366 bacterial genera identified with 16s rRNA sequencing, 19 accounted for ≥ 1% in relative abundance (Table 3). The 5 most abundant genera were *Bifidobacterium* (11.5%), *Bacteroides* (11%), *Streptococcus* (9.8%), *Enterobacter* (7.3%), and *Lachnospira* (5.9%).

**Table 3:** Relationship Between 19 Genera With Relative Abundance ≥1% and Recurrence-free Survival and Overall Survival

No	Bacterial genera	Cutoff values based on RA, % (IQR)	*P* value of log-rank test between high and low RA		Univariable analysis in Cox proportional hazards model			
			RSF	OS	RFS		OS	
					HR (95% CI)	*P* value	HR (95% CI)	*P* value
1	*Bifidobacterium*	4.0 (0.9–17.5)	**.037**	**.032**	0.41 (0.17–0.98)	**.046**	0.94 (0.84–1.00)	.064
2	*Bacteroides*	8.4 (2.7–14.4)	.95	.39	0.97 (0.42–2.26)	.95	1.81 (0.46–7.12)	.39
3	*Streptcoccus*	5.3 (1.0–17.4)	.63	.17	0.82 (0.36–1.87)	.63	2.50 (0.65–9.70)	.18
4	*Enterobacter*	2.1 (0.2–13.6)	.62	.36	0.82 (0.36–1.86)	.63	0.56 (0.16–1.99)	.37
5	*Lachnospira*	4.8 (2.2–6.6)	.34	.84	1.48 (0.65–3.39)	.35	1.14 (0.33–3.98)	.84
6	*Enterococcus*	0.2 (0.02–1.6)	.59	.23	1.80 (0.73–4.35)	.21	0.50 (0.14–1.80)	.29
7	*Akkermansia*	0.02 (0–0.9)	.42	.50	1.59 (0.70–3.63)	.27	1.55 (0.44–5.50)	.50
8	*Blautia*	2.0 (0.7–3.7)	.25	.90	0.57 (0.25–1.31)	.19	0.92 (0.26–3.19)	.90
9	*Lactobacillus*	0.1 (0–1.4)	.45	.45	0.73 (0.32–1.68)	.46	1.62 (0.46–5.77)	.46
10	*Prevotella*	0.004 (0–0.1)	.22	.31	0.61 (0.26–1.38)	.23	0.52 (0.15–1.87)	.32
11	*Faecalibacterium*	0.6 (0.1–3.8)	.30	**.025**	1.53 (0.67–3.50)	.31	1.17 (0.95–1.42)	.13
12	*Megamonas*	0.0 (0–0.007)	.36	.49	1.49 (0.63–3.52)	.37	1.57 (0.44–5.57)	.49
13	*Collinsella*	1.2 (0–2.6)	.64	.83	1.21 (0.53–2.76)	.65	1.15 (0.33–3.98)	.83
14	*Clostridium_sensu_stricto_1*	0.03 (0–1.1)	.92	.58	0.99 (0.39–2.54)	.99	0.99 (0.26–3.82)	.99
15	*Dickeya*	0.2 (0.02–0.8)	.26	.78	1.59 (0.69–3.62)	.27	0.84 (0.24–2.97)	.78
16	*Subdoligranulum*	0.6 (0.02–1.8)	.31	1.00	0.44 (0.17–1.13)	.09	1.00 (0.29–3.46)	1.00
17	*Parabacteroides*	1.0 (0.2–1.8)	.77	.26	1.48 (0.55–4.01)	.44	2.15 (0.55–8.38)	.27
18	*Alistipes*	0.5 (0.1–1.1)	1.00	.24	0.58 (0.25–1.37)	.22	2.20 (0.57–8.50)	.25
19	*Eubacterium_hallii_group*	0.2 (0–1.7)	.41	.34	1.40 (0.62–3.20)	.41	1.83 (0.52–6.51)	.35

Bold indicates statistically significant differences with *P* values less than .05.

CI, confidence interval; HR, hazard ratio; IQR, interquartile range; OS, overall survival; RA, relative abundance; RSF, recurrence-free survival.

### Comparison of RFS and OS Between High- and Low-relative Abundance of Each Genus

**Figure 2:**
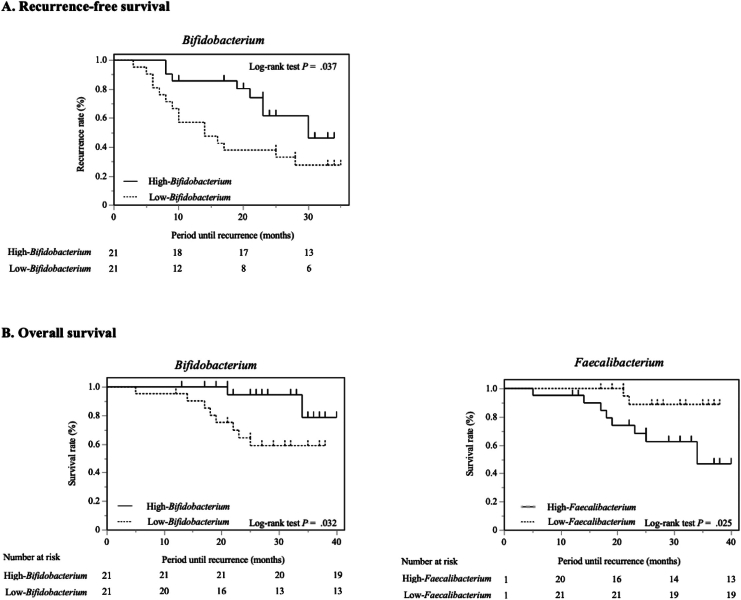
Kaplan–Meier estimates of recurrence-free survival (A) and overall survival (B).

To explore candidate microbial genera for predicting postoperative RFS and OS, we compared RFS and OS between patients with high and low relative abundances of each of the 19 genera using Kaplan–Meier curves and log-rank tests. The results are presented in Table 3, Figure 2, and Figures A1, A2, and A3.

As shown in Figure 2A, patients with a high relative abundance of *Bifidobacterium* (≥4.0%) showed extended RFS compared to those with a low relative abundance of *Bifidobacterium* (<4.0%) (*P* = .037). In the comparison of RFS with the log-rank test between high and low relative abundance levels of microbes, there were no statistical differences in the remaining 18 microbes other than *Bifidobacterium* (Table 3 and Figure A1). In the Cox proportional hazards model, multivariable analysis including *Bifidobacterium* revealed high-*Bifidobacterium* (hazard ratio [HR] 0.37; 95% confidence interval [CI] 0.14–0.97, *P* = .042) and pathological metastasis of lymph node (HR 3.12, 95% CI 1.21–7.99, *P* = .018) were significantly associated with RFS (Table 4).

**Table 4:** Univariable and Multivariable Analyses of Risk Factors for Postoperative Recurrence

	Univariable analysis		Multivariable analysis	
	Hazard ratio (95% CI)	*P* value	Hazard ratio (95% CI)	*P* value
Male	1.19 (0.51–2.74)	.69	0.53 (0.20–1.38)	.19
Age	0.81 (0.36–1.85)	.62	0.88 (0.38–2.03)	.77
Body mass index	0.98 (0.87–1.03)	.67		
Prognostic nutritional index	0.96 (0.89–1.05)	.37		
Neutrophil-to-lymphocyte ratio	1.90 (0.26–10.57)	.49		
Albumin	0.65 (0.30–1.61)	.30		
CA19-9	1.48 (0.63–3.50)	.37		
CA19-9 decreased ≥50% after NAC	0.81 (0.33–1.98)	.65		
Pancreatic head tumor	1.17 (0.46–2.97)	.74		
Resectable PDAC	0.56 (0.18–1.64)	.28		
Relative dose intensity of NAC	1.00 (0.98–1.02)	.79		
Evans grade ≥2	0.91 (0.31–2.70)	.87		
Pathological size of PDAC	1.84 (0.68–4.97)	.23		
Pathological metastasis of lymph node	2.98 (1.22–7.28)	.016	3.12 (1.21–7.99)	**.018**
Postoperative complications	0.64 (0.24–1.75)	.39		
High-*Bifidobacterium*	0.41 (0.17–0.98)	.046	0.37 (0.14–0.97)	**.042**

CI, confidence interval; NAC, neoadjuvant chemotherapy; PDAC, pancreatic ductal adenocarcinoma.

As to OS, the Kaplan–Meier curves demonstrated that patients with high-relative abundance of *Bifidobacterium* (≥4.0%) and those with low-relative abundance of *Faecalibacterium* (<0.6%) showed significantly more favorable OS compared to patients with low-relative abundance of *Bifidobacterium* (<4.0%) and high-relative abundance of *Faecalibacterium* (≥0.6%), respectively (*Bifidobacterium*; *P* = .032, *Faecalibacterium*; *P* = .025) (Figure 2B). For the remaining 17 genera, there were no significant differences in OS between the high and low relative abundance groups (Table 3 and Figure A2). In the Cox proportional hazards model, multivariable analysis showed no factors associated with OS (*Bifidobacterium*; HR 0.23, 95% CI 0.03–1.50, *P* = .18) (Table 5). However, CIs were generally wide and were compatible with both a substantially lower and substantially higher mortality risk. In particular, pathological metastasis of lymph node showed substantially wide CI.

**Table 5:** Univariable and Multivariable Analyses of Risk Factors for Overall Survival

	Univariable analysis		Multivariable analysis	
	Hazard ratio (95% CI)	*P* value	Hazard ratio (95% CI)	*P* value
Male	1.28 (0.36–4.57)	.71	0.28 (0.06–1.31)	.11
Age	0.94 (0.27–3.27)	.85	1.01 (0.95–1.09)	.66
Body mass index	0.95 (0.79–1.13)	.59		
Prognostic nutritional index	0.88 (0.78–0.99)	.040	0.96 (0.83–1.11)	.60
Neutrophil-to-lymphocyte ratio	0.58 (0.02–8.29)	.72		
Albumin	0.45 (0.15–1.63)	.17		
CA19-9	0.66 (0.19–2.31)	.52		
CA19-9 decreased ≥50% after NAC	0.17 (0.02–1.39)	.04	0.16 (0.02–1.76)	.14
Pancreatic head tumor	3.93 (0.50–31.13)	.12		
Resectable PDAC	0.31 (0.08–1.20)	.12		
Relative dose intensity of NAC	0.98 (0.95–1.02)	.25		
Evans grade ≥2	0.74 (0.15–3.54)	.70		
Pathological size of PDAC	4.05 (0.51–32.01)	.047	1.03 (0.95–1.12)	.46
Pathological metastasis of lymph node	9.57 (1.21–75.63)	.005	11.11 (0.97–126.95)	.053
Postoperative complications	0.62 (0.13–2.96)	.53		
High-*Bifidobacterium*	0.94 (0.84–1.00)	.064	0.23 (0.03–1.50)	.18
High-*Faecalibacterium*	1.17 (0.95–1.42)	.13		

CI, confidence interval; NAC, neoadjuvant chemotherapy; PDAC, pancreatic ductal adenocarcinoma.

**Figure 3:**
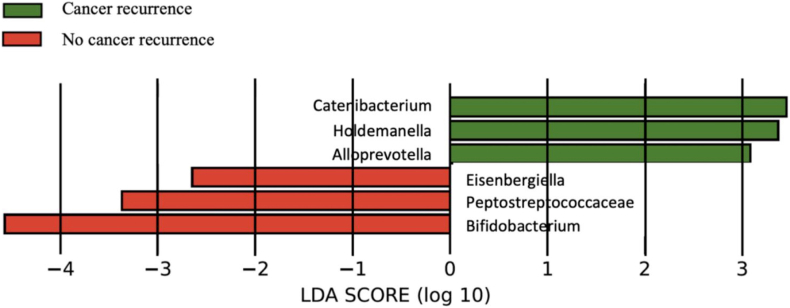
LEfSe analysis identifying 6 genera that is related to cancer recurrence Histogram of the linear discriminant analysis (LDA) scores for relevance to cancer recurrence within 12 months after surgery.

LEfSe analysis identified *Catenibacterium*, *Holdemanella*, *Alloprevotella*, *Eisenbergiella*, *Peptostreptococcaceae*, and *Bifidobacterium,* as microbial genera that were significantly relevant to cancer recurrence within 12 months after surgery (Figure 3). Of these 6 genera, *Bifidobacterium* had the highest Linear Discriminant Analysis score.

### Association Between Cancer Recurrence and Diversity of Gut Microbiota and Relative Abundance of the Relative Abundance of *Bifidobacterium* Genus

We conducted an additional analysis to explore the differences in diversity and the relative abundance of *Bifidobacterium* genus between patients with and without cancer recurrence within 12 months after surgery (Figure A4A and A4B). There were no significant differences in alpha diversity,such as Chao-1 index (*P* = .87) and Shannon's diversity index (*P* = .85), and beta diversity (*P* = .43) at the time of PDAC diagnosis. Meanwhile, the relative abundance of *Bifidobacterium* before treatment was higher in patients without recurrence (10.7%) than that in patients with recurrence (1.9%) (*P* = .048) (Figure A5).

## Discussion

This is the first report on the relationship between prognosis after resection of R/BR-PDAC and gut microbiota. Patients with a higher relative abundance of *Bifidobacterium* had a significantly favorable RFS. Furthermore, multivariable analysis using the Cox proportional hazards model demonstrated that high-*Bifidobacterium* were associated with a favorable RFS. From LEfSe analysis, *Bifidobacterium* was involved with early recurrence (≤12 months) after resection. The results of the current study suggest that the presence of the genus *Bifidobacterium* at PDAC diagnosis potentially serves as a microbial marker for predicting PDAC recurrence after pancreatectomy following NAC.

In clinical practice, surgical resection after NAC therapy has become the mainstream treatment strategy for R/BR-PDAC.^22^ Under such circumstances, several studies have reported biomarkers for predicting the prognosis of patients with PDAC receiving preoperative chemotherapy. Yamada et al. reported that patients with normalized CA19-9 levels after NAC therapy had significantly longer progression-free survival than those without normalized CA19-9 levels.^23^ Akita et al. reported that high CA19-9 levels (>200 U/L) after NAC were associated with poor prognosis in patients with R/BR-PDAC.^24^ Ono et al. reported that high CA19-9 levels (>120), and lymph node metastasis, tumor diameter > 2 cm, diabetes, and retroperitoneal invasion were risk factors for early recurrence.^25^ Tezuka et al. reported that the prognostic factors for patients with BR-pancreatic cancer who underwent pancreatectomy after NAC included decreased CA19-9 levels after NAC, the presence of lymph node metastasis, and NLR.^26^ Similar to previous reports,^25^^,^^26^ we confirmed that lymph node metastasis was associated with poor RFS, although there was no association between RFS and CA19-9 levels measured before and after NAC. The relative abundance of fecal *Bifidobacterium* at PDAC diagnosis was significantly associated with RFS. Despite growing positive evidence for NAC in R/BR-PDAC, in clinical practice, certain patients experience disease progression during NAC, resulting in the inability to achieve curative surgical resection.^27^ Considering our previous study showing the relationship between fecal *Bifidobacterium* and the pathological response to NAC in R/BR-PDAC,^11^ quantifying the genus *Bifidobacterium* at the diagnosis of PDAC is advantageous because it allows us to predict the therapeutic efficacy and prognosis before the introduction of NAC.

A few studies have reported the relationship between gut microbiota and the prognosis of PDAC after treatment. Analysis of the fecal metagenome by Kharofa et al. demonstrated that the relative abundance of *Faecalibacterium prausnitzii* is significantly associated with disease free survival in patients with PDAC.^28^ Nagata et al. examined microbial species relevant to favorable and poor prognosis of PDAC using shotgun metagenomic analysis of fecal samples collected from patients with PDAC and non-PDAC controls.^17^ Microbial species with a higher abundance were significantly associated with a favorable prognosis, including unknown *Alistipes, F. prausnitzii*, and *Enterobacteriaceae* species in the gut. In our study, a higher abundance of *Bifidobacterium* was associated with a better RFS; however, the microbial genera were not identified in these 2 studies. The reasons for this discrepancy were that the race of the patients, stage of PDAC, and treatment differed. Race is a key determinant of the human gut microbiome, and PDAC has been reported to have different gut microbiota depending on the stage of progression.^29^ In a study focusing on Japanese patients with R/BR-PDAC who underwent NAC followed by curative resection, we identified *Bifidobacterium* as a microbial marker associated with PDAC prognosis.

Previous studies have suggested that *Bifidobacterium* has beneficial effects on antitumor immunity. The results of an animal experiment by Sivan et al. showed *Bifidobacterium* could augment dendritic cell function, promote the infiltration of tumor-specific CD8^+^ T cells into the tumor microenvironment, regulate the activation of cytokine receptors to produce interferon-γ, and then promote the growth of monocytes.^30^ Therefore, *Bifidobacterium* may positively influence the response to chemotherapy and checkpoint inhibitors in patients with malignant neoplasms. Supplementation with CBM588, a bifidogenic live bacterial product, has improved clinical outcomes in patients with metastatic renal cell carcinoma receiving nivolumab and ipilimumab.^31^ In addition, intestinal microflora can regulate the composition of the microbiota and reduce adverse events caused by chemotherapeutic drugs, such as diarrhea and mucositis.^32^ The administration of *Bifidobacterium* reduces the frequency of fever and the need for antibiotics.^33^ In the current study, despite similar clinical backgrounds, including chemotherapy-associated factors (regimen, RDI, and adverse events), patients with a high relative abundance of *Bifidobacterium* had a more favorable prognosis without recurrence, suggesting that commensal *Bifidobacterium* enhances the tumor-suppressive effect of NAC on PDAC. From these results, in chemotherapy of PDAC, administration of probiotics, including *Bifidobacterium,* may reinforce the therapeutic effects of chemotherapy in patients with proven lower *Bifidobacterium* relative abundance before treatment, although the molecular biological mechanism of linkage between the gut microbiome and outcome of PDAC remains poorly understood. Therefore, further studies are needed.

This study had certain limitations. First, the sample size was small because this was a single-center study. Second, the follow-up period after surgery was relatively short, potentially leading to the failure of the multivariable analysis to identify factors associated with OS. Further prolongation of the follow-up period may clarify the relationship between the gut microbiome and OS in patients receiving NAC. Third, the multiple tests for the 19 genera potentially cause false-positive results because false discovery rate correction was not carried out for log-rank tests. The finding that *Bifidobacterium* was statistically associated with RFS was should be validated in large, well-designed studies with sufficient follow-up period. Fourth, we evaluated the gut microbiome only during PDAC diagnosis, not after NAC or pancreatic resection. Although chemotherapy is known to influence the gut microbiome,^17^ microbial data must be following chemotherapy or during the development of severe adverse events. Microorganisms in the gut may impact the incidence of adverse events during NAC with GS therapy and adjuvant therapy with S-1 in the long-term, which potentially influences prognosis. Future research with adding the microbiome data after NAC post-NAC and during the development of adverse events are required. Finally, our cohort included only the Japanese population, possibly leading to a selection bias.

## Conclusion

In conclusion, the relative pretreatment fecal levels of *Bifidobacterium* could be a predictor of postoperative recurrence in patients with R/BR-PDAC treated with NAC.

## Supplementary materials

Supplementary Figure 1 Comparison of RFS between patients with high and low relative abundances of each of the 19 genera. The genus numbers apply to those describes in Table 3. Supplementary Figure 2. Comparison of OS between patients with high and low relative abundances of each of the 19 genera. The genus numbers apply to those describes in Table 3. Supplementary Figure 3. Forest plot for RFS and OS of 19 genera Supplementary Figure 4. Comparison of microbial diversity between patients with and without cancer recurrence within 12 months after surgery Supplementary Figure 5. Relationship between cancer recurrence within 12 months after surgery and the relative abundance of *Bifidobacterium* before treatment

Extended PDF
