# Supplementary material for: Fecal Bifidobacterium Serves as a Predictor of Postoperative Recurrence After Neoadjuvant Chemotherapy in Pancreatic Cancer
Source: Gastro Hep Adv. 2025 Aug 28;5(1):100779. doi: 10.1016/j.gastha.2025.100779 (PMC12546966; doi:10.1016/j.gastha.2025.100779)

Supplementary Figure 1

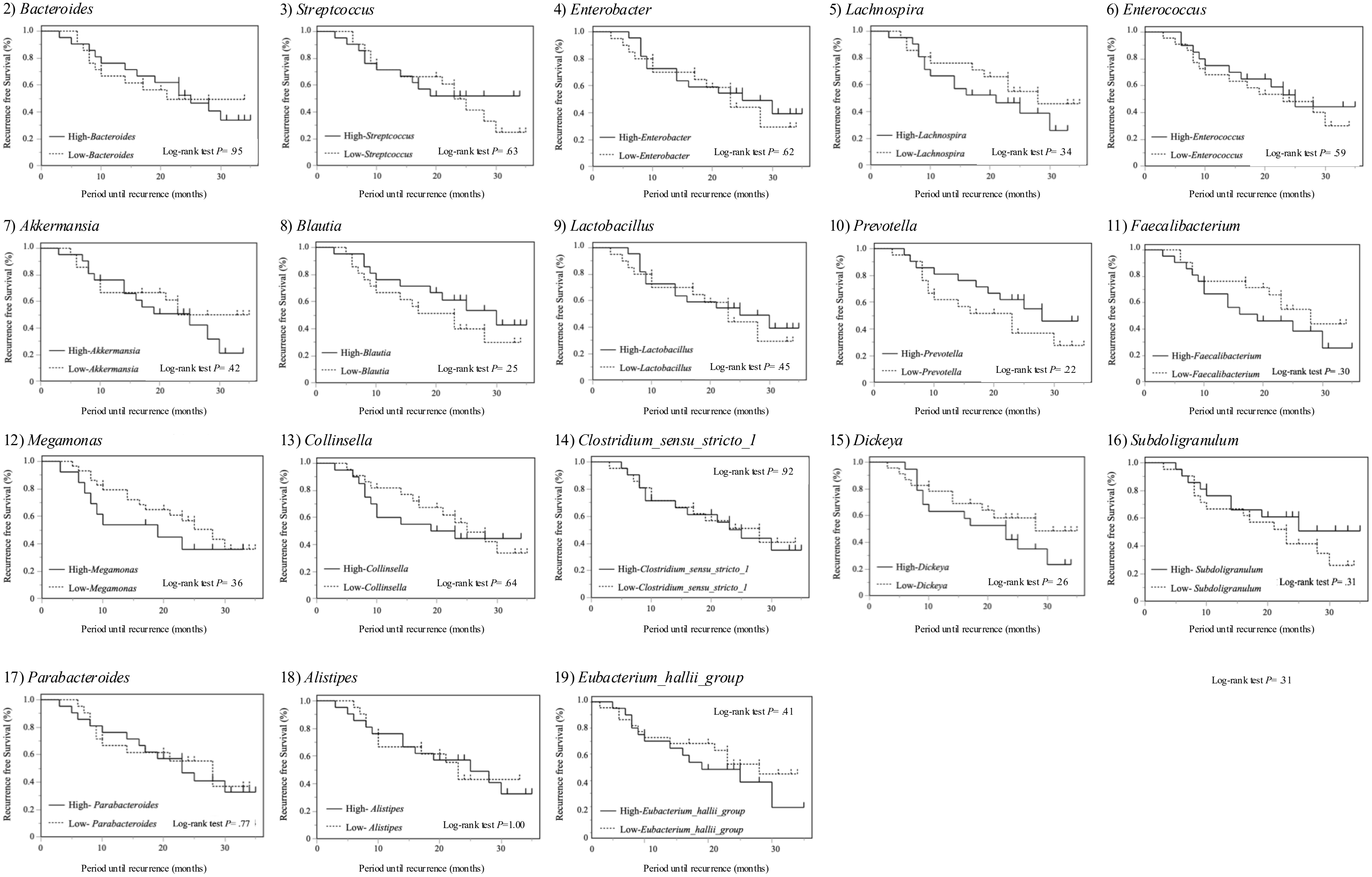

The genus numbers apply to those describes in Table 3.

Supplementary Figure 2

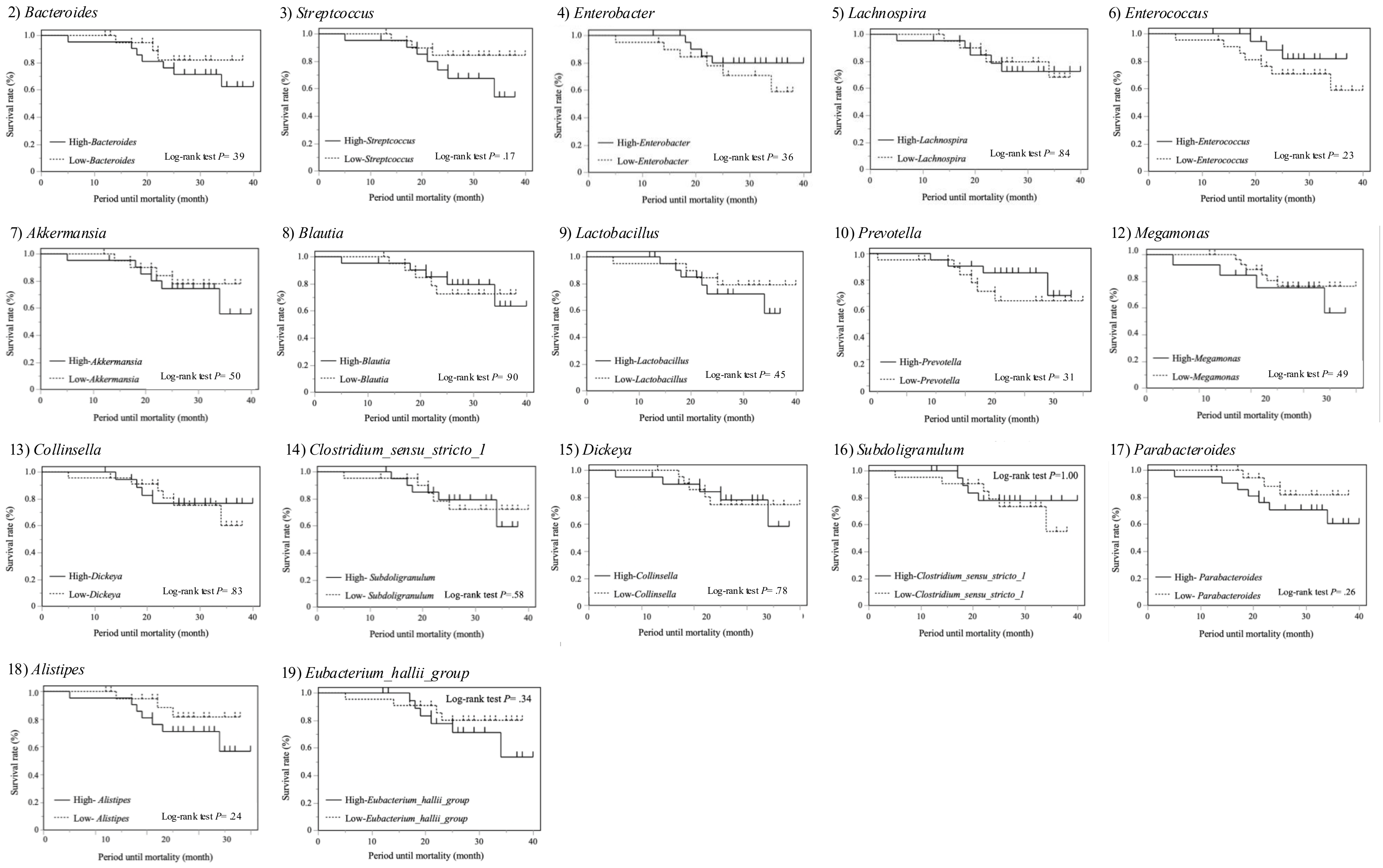

The genus numbers apply to those describes in Supplementary Table 3.

Supplementary Figure 3

A. Forest plot for recurrence-free survival

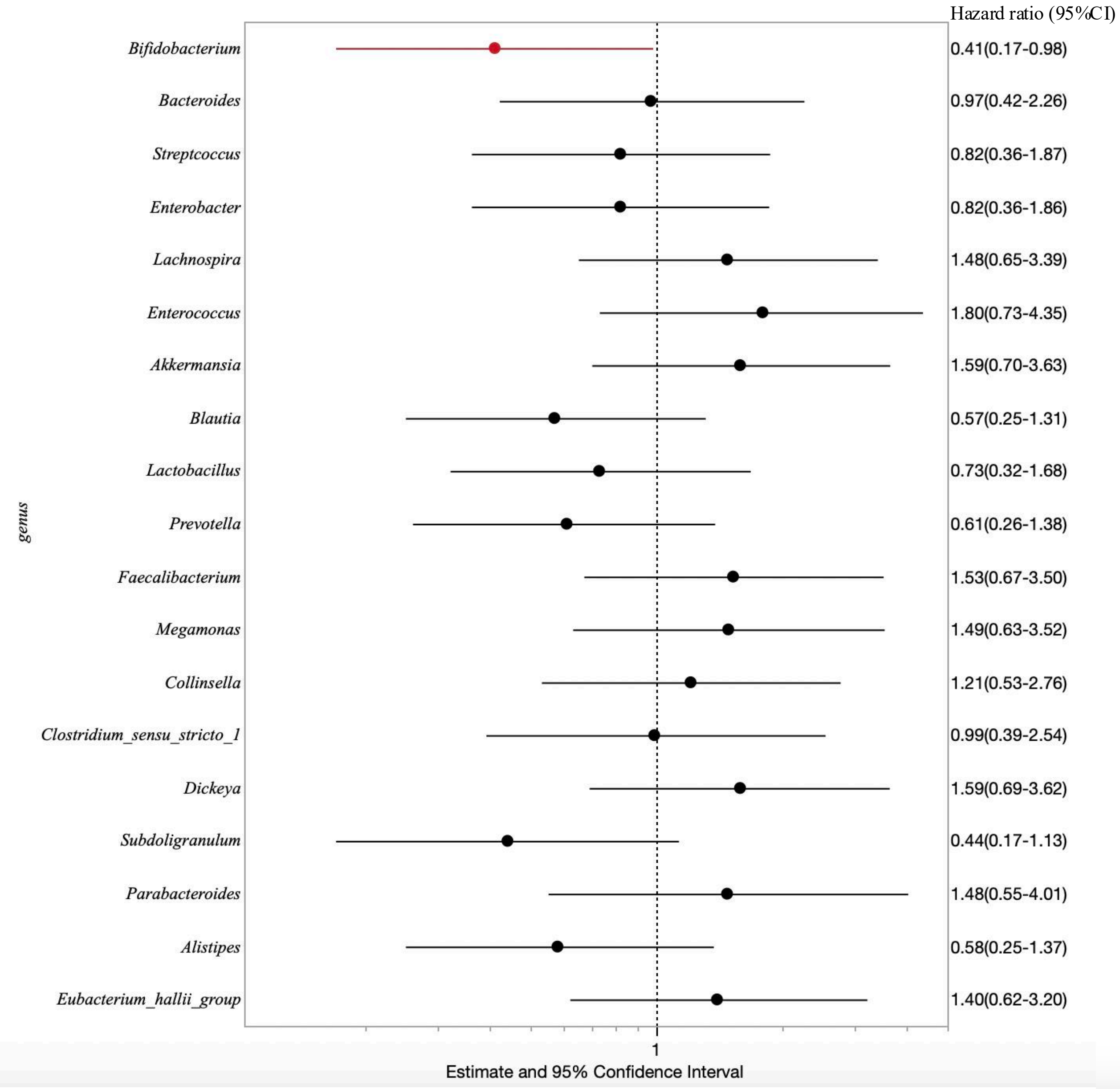

B. Forest plot for overall survival

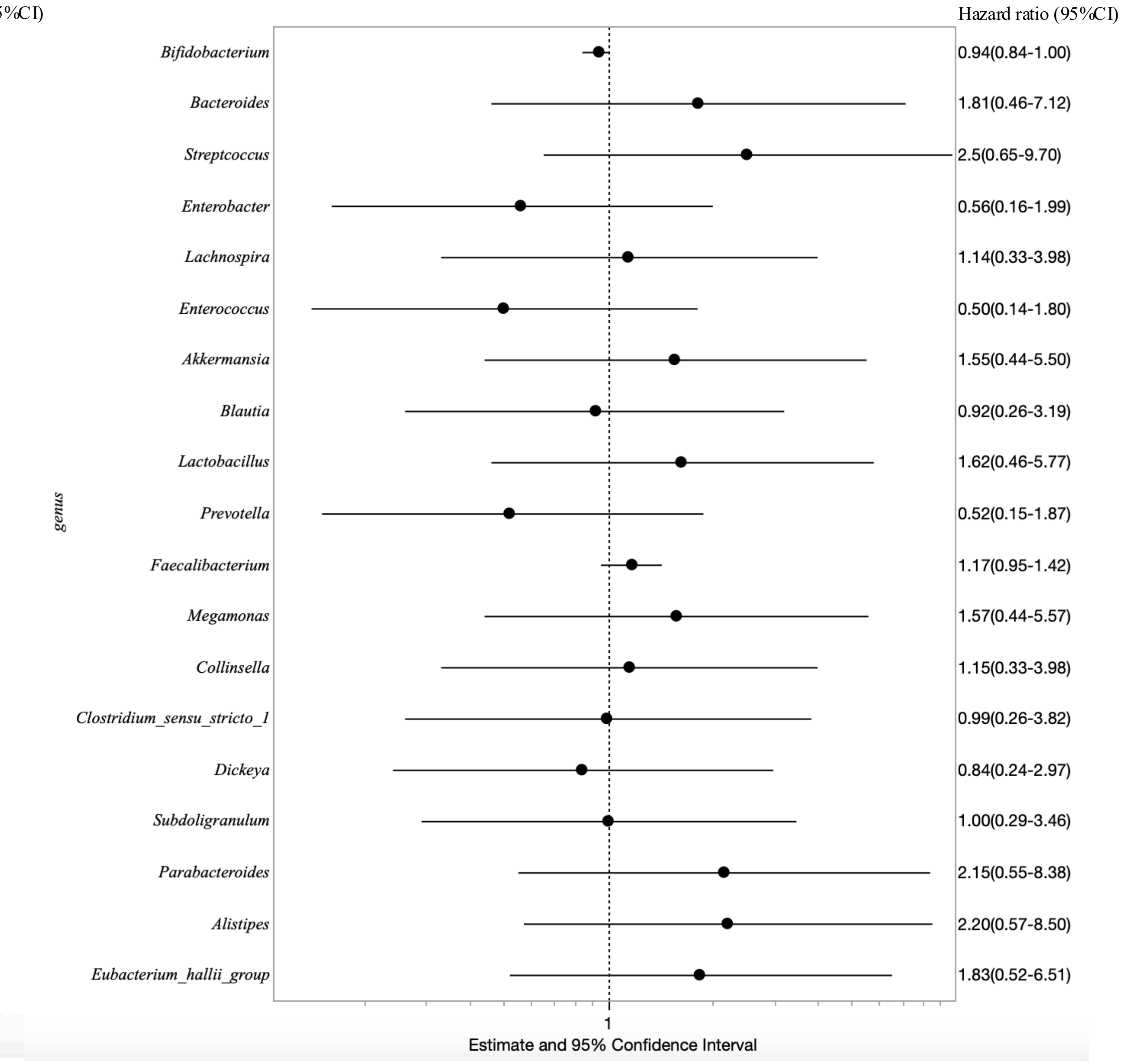

Supplementary Figure 4

A. Comparison of alpha diversity between patients with and without recurrence within 12 months after surgery

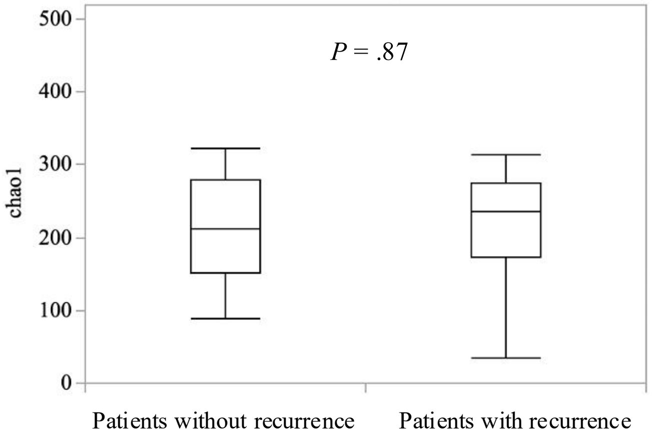

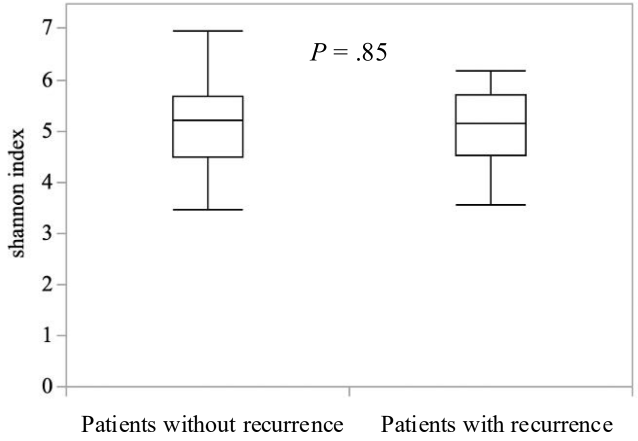

B. Comparison of beta diversity between patients with and without recurrence within 12 months after surgery

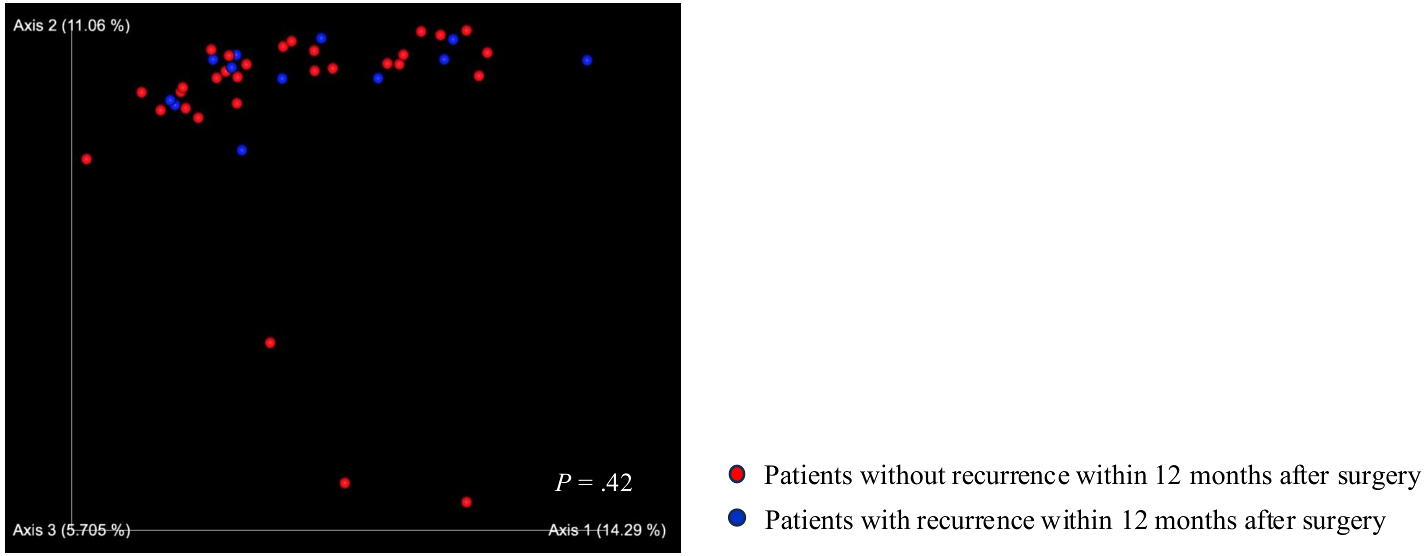

Supplementary Figure 5

Relationship between cancer recurrence within 12 months after surgery and the relative abundance of *Bifidobacterium* before treatment

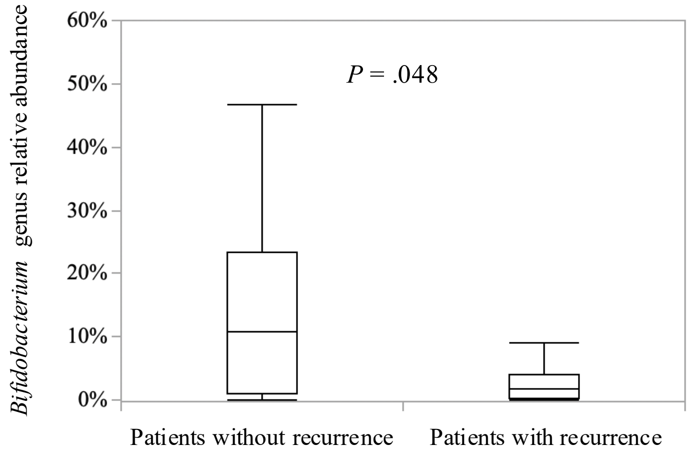

Supplement: Supplementary Figure 1 — Comparison of RFS between patients with high and low relative abundances of each of the 19 genera. The genus numbers apply to those describes in Table 3. Supplementary Figure 2. Comparison of OS between patients with high and low relative abundances of each of the 19 genera. The genus numbers apply to those describes in Table 3. Supplementary Figure 3. Forest plot for RFS and OS of 19 genera Supplementary Figure 4. Comparison of microbial diversity between patients with and without cancer recurrence within 12 months after surgery Supplementary Figure 5. Relationship between cancer recurrence within 12 months after surgery and the relative abundance of Bifidobacterium before treatment [file mmc1.pdf]
